# The Serum Treatment of Exophthalmic Goitre

**Published:** 1907-12

**Authors:** 


### The Serum Treatment of Exophthalmic Goitre.

Harriet C. B. Alexander, *American Practitioner and News,* August, 1907, discusses the subject and reports thirteen cases. Four principal theories of the disease have been advanced: (1) That it is due to disease of the sympathetic nervous system; (2) that the seat of the malady is the medulla oblongata; (3) that it is primarily a disease of the thryoid gland; and (4) that it is a neurosis.

Modern therapeutic measures have been largely based on the thyroid theory. The results of partial strumectomy indicate that the successful removal of a portion of the thyroid gland can lead to cure or to definite amelioration of the condition. On the theory that the thyroid secretion normally neutralises certain general metabolic poisons in the body, Moebius and others conceived of treating cases of exophthalmic goitre, in which there is presumably an excess of thyroid secretion in the body, by introducing subcutaneously, or by the mouth, the serum of thyroidectomised animals. It was hoped that the non-neutralised general metabolic poisons of such animals would nullify the toxic effect of the excessive thryoid secretion. As to the treatment, experience has shown the great importance of general measures: complete rest for a time, fresh air, careful diet, mild balneotherapy, etc.

The name thyroidectin has been given to a preparation obtained under the aseptic precautions from the blood of animals from which the thyroid glands have been removed, and which is exhibited as a reddish-brown powder contained in capsules, usually five grains each. Carefully conducted clinical trials seem to show that thyroidectin can be depended upon to control the characteristic symptoms of exophthalmic goitre. In most cases the patient experiences much relief from the restlessness, tremors, insomnia and other nervous symptoms so frequently present, and a gradual lessening of the frequency of the pulse rate, decrease in the size of the glands, and a diminution of the exophthalmos, with an increase of weight and a much better condition generally. The dose of thyroidectin seems to be one or more capsules after each meal, according to the judgment of the physician and the reaction of the patient.

In nine of the author's thirteen cases the size of the gland was materially reduced, and in every case improvement was observed with respect to one or more of the symptoms.

---

THE MISUSE OF THE VOICE AND ITS CURE.—N. J. Poock Van Baggen of The Hague describes the exercises needed to correct the false use of the voice after proper breathing has been learned. The mouth must be opened widely. The back of the tongue becomes too much developed and the tip must be exercised instead. To cure imperfect articulation the exercises must develop different groups of muscles separately. The author describes exercises for the development of the tip of the tongue by articulation of vowels and consonants.—*Medical Record*, November 23, 1907.

